# DC-STAMP and OC-STAMP cooperatively regulate osteoclast and foreign body giant cell cell–cell fusion

**DOI:** 10.1007/s00774-025-01667-y

**Published:** 2025-12-10

**Authors:** Fuka Homma, Ryotaro Iwasaki, Makoto Tateyama, Tomoya Soma, Mayu Morita, Makiko Kashio, Taneaki Nakagawa, Takeshi Miyamoto

**Affiliations:** 1 Department of Dentistry and Oral Surgery, Keio University School of Medicine, 35 Shinano-Machi, Shinjuku-Ku, Tokyo, 160-8582 Japan; 2 Department of Orthopedic Surgery, Faculty of Life Sciences, Kumamoto University, 1-1-1 Honjo, Chuo-Ku, Kumamoto, 860-8556 Japan; 3 Department of Cell Physiology, Faculty of Life Sciences, Kumamoto University, 5-1 Oe, Chuo-Ku, Kumamoto, 862-0973 Japan

**Keywords:** DC-STAMP, OC-STAMP, Osteoclast, Foreign body giant cell, Cell–cell fusion

## Abstract

**Introduction:**

Osteoclasts and foreign body giant cells (FBGCs) are multi-nuclear cells established by fusion of their mono-nuclear forms. Multi-nucleation via cell–cell fusion is a common characteristic of osteoclasts and FBGCs, and Dendritic Cell-Specific Transmembrane Protein (DC-STAMP) and Osteoclast Stimulatory Transmembrane Protein (OC-STAMP) are both required for the process. Thus, it is thought that DC-STAMP and OC-STAMP interaction likely induces this fusion, but details of these mechanisms are not clear.

**Materials and methods:**

We crossed DC-STAMP knockout (KO) with OC-STAMP KO mice to obtain DC-STAMP/OC-STAMP doubly deficient (DKO) mice. Osteoclasts and FBGC common progenitors were isolated from wild-type (WT), DC-STAMP KO, OC-STAMP KO or DKO mice. We then set up 4 co-culture systems: (1) WT with DC-STAMP KO cells, (2) WT with OC-STAMP KO cells, (3) WT with DKO cells, and (4) DC-STAMP KO with OC-STAMP KO cells to induce osteoclast or FBGC formation. We evaluated osteoclast and FBGC formation by TRAP and May–Gruenwald Giemsa staining, respectively. Finally, we performed bone morphometric analysis of WT, DC-STAMP KO, OC-STAMP KO, and DKO mice.

**Results:**

Cell–cell fusion occurs normally in co-cultures of DC KO with WT cells, OC KO with WT cells, and DKO with WT cells in both osteoclast and FBGC-inducing conditions. By contrast, co-cultures of DC-STAMP KO with OC-STAMP KO cells did not show cell–cell fusion. Bone morphometric analysis revealed enhanced bone formation in DKO mice.

**Conclusion:**

DC-STAMP and OC-STAMP cooperate to promote osteoclast and FBGC cell–cell fusion. DC-STAMP and OC-STAMP doubly deficient mice exhibit increased osteogenesis.

**Supplementary Information:**

The online version contains supplementary material available at 10.1007/s00774-025-01667-y.

## Introduction

Osteoclasts, which undergo terminal differentiation from monocyte-macrophage line age cells [1], are unique bone-resorbing cells in the body. Various molecules such as macrophage fusion receptor (MFR), E-Cadherin, and CD44 reportedly regulate osteoclast fusion [2–4], but thus far no factor has been identified as required for cell–cell osteoclast fusion based on analysis of gene knockout mice. Previously, we identified Dendritic Cell-Specific Transmembrane Protein (DC-STAMP) and generated DC-STAMP gene knockout (KO) mice [5]. DC-STAMP KO mice exhibited complete loss of osteoclast cell–cell fusion, while expression of osteoclast differentiation markers in DC-STAMP-deficient mono-nuclear osteoclasts was comparable to that seen in multi-nuclear osteoclasts from WT mice [5], indicating that DC-STAMP is specifically required for osteoclast fusion rather than differentiation. Interestingly, co-culture of osteoclast progenitors derived from DC-STAMP KO mice with those from WT mice resulted in multi-nuclear osteoclast formation by fusion of both cells [5]. We conclude that cell–cell fusion is induced in osteoclast progenitor cells derived from DC-STAMP KO mice by interaction of factors that interact with DC-STAMP expressed in DC-STAMP KO cells with DC-STAMP expressed in osteoclast progenitor cells derived from WT mice.

Osteoclast Stimulatory Transmembrane Protein (OC-STAMP) was identified in osteoclasts after DC-STAMP was identified [6] and OC-STAMP-deficient mice were established [7]. OC-STAMP-deficient mice also exhibit complete loss of osteoclast cell–cell fusion, while expression of osteoclast differentiation markers in mono-nuclear osteoclasts derived from OC-STAMP-deficient mice is comparable to that seen in multi-nuclear osteoclasts from WT mice [7], indicating that OC-STAMP is specifically required for osteoclast fusion rather than differentiation. Moreover, co-cultivation of osteoclast progenitor cells derived from OC-STAMP KO mice with osteoclast progenitors from WT mice induces multi-nuclear osteoclast formation via fusion of both cells [7]. Thus, DC-STAMP KO and OC-STAMP KO mice exhibit identical phenotypes in terms of osteoclast fusion, suggesting that osteoclast fusion proceeds via DC-STAMP/OC-STAMP interaction.

After it was cloned, the *DC-STAMP* gene, which is expressed in dendritic cells and IL-4-stimulated macrophages, was found to encode a multiple transmembrane domain protein [8, 9], while OC-STAMP has been identified in osteoclasts and shares features similar to other multiple transmembrane domain molecules, such as DC-STAMP [10]. However, whether DC-STAMP/OC-STAMP interaction is required for osteoclast fusion remained unclear.

Induction of foreign body giant cells (FBGCs), which are also monocyte-macrophage lineage multi-nuclear cells like osteoclasts [11], occurs in response to foreign bodies in the body. FBGCs differentiate from osteoclast/FBGC common progenitors and form multi-nuclear cells by fusion of mono-nuclear cells, as is seen in osteoclasts [11]. Interestingly, multi-nuclear FBGC formation by cell–cell fusion is completely lost in either DC-STAMP KO or OC-STAMP KO mice [5, 7]. However, again, whether DC-STAMP/OC-STAMP interaction is required for FBGC fusion remains to be elucidated.

Here, to assess the role of DC-STAMP/OC-STAMP interaction in the fusion of osteoclasts and FBGCs, we generated DC-STAMP/OC-STAMP doubly deficient (DKO) mice by crossing DC-STAMP KO with OC-STAMP KO mice. We then co-cultivated osteoclast and FBGC common progenitor cells derived from DKO mice with comparable cells from WT mice. We also analyzed cell–cell fusion of osteoclasts or FBGCs by co-culturing osteoclast/FBGC common progenitors from DC-STAMP KO mice with osteoclast/FBGC common progenitors from OC-STAMP KO mice. We also performed bone morphometric analysis of DKO mice.

## Materials and methods

### Mice

WT mice were purchased from CLEA Japan, Inc. (Tokyo, Japan). Mice deficient in either DC-STAMP or OC-STAMP were both generated previously [5, 7]. Animals were maintained under specific pathogen-free conditions in animal facilities certified by the Kumamoto University Center for Animal Resources and Development or Keio University Institutional Animal Care, and animal protocols were approved by those committees.

### Analysis of skeletal morphology

Bone mineral density (BMD, mg/cm^2^) was analyzed using dual energy X-ray absorptiometry (DXA) in 8-week-old WT, DC-STAMP KO, OC-STAMP KO, or DKO mice. Bone morphometric analysis was performed to determine bone volume/tissue volume (BV/TV, %), trabecular thickness (Tb.Th, µm), trabecular number (Tb.N, /mm), trabecular separation (Tb.Sp, µm), osteoclast surface/bone surface (Oc.S/BS, %), osteoblast surface/bone surface (Ob.S/BS, %), eroded surface/bone surface (ES/BS, %), number of osteoclasts (N.Oc/B.Pm per 100 mm), mineral apposition rate (MAR, µm/d), and bone formation rate/bone surface (BFR/BS, µm^3^/µm^2^/d) in 8-week-old WT, DC-STAMP KO, OC-STAMP KO, or DKO mice, as previously described [12, 13],.

### Osteoclast and foreign body giant cell culture

Osteoclasts and FBGCs were induced as described [5, 7]. Briefly, mouse bone marrow mono-nuclear cells were collected and cultured 4 days in the presence of M-CSF (50 ng/mL), and then M-CSF-dependent progenitors were isolated as osteoclasts/FBGC common progenitor cells. For osteoclast induction, osteoclasts/FBGC common progenitor cells were cultured in the presence of M-CSF (50 ng/mL) and RANKL (25 ng/mL) for 3 or 4d and stained by tartrate-resistant acid phosphatase (TRAP), and then multi-nuclear TRAP-positive cells were scored as osteoclasts. For FBGC induction, osteoclasts/FBGC common progenitor cells were cultured 3d with G-MCSF (50 ng/mL) and IL-4 (50 ng/mL) and stained with May–Grunwald Giemsa, and then multi-nuclear FBGCs were counted. In some cultures, osteoclasts/FBGC common progenitor cells derived from WT, DC-STAMP KO, OC-STAMP KO, or DKO mice were mixed and cultured in combinations described in the Results and then evaluated for osteoclast or FBGC formation in each culture condition.

### Immunofluorescent staining

Osteoclasts or FBGCs cultured as above were fixed with 4% paraformaldehyde and stained with the anti-GFP antibody (1:1000 MBL Co., Tokyo Japan), followed by Alexa488-conjugated anti-rabbit IgG antibody (1:200 Thermo Fisher scientific, Waltham MA) in the presence of cellstain^®^-DAPI (Fujifilm, Tokyo, Japan) to stain nuclei.

### Real-time PCR and RT-PCR analysis

Total RNA was isolated from osteoclasts or FBGCs using an RNeasy mini kit (Qiagen, Hilden, Germany), as described above. Single-stranded complementary DNAs (cDNAs) were synthesized with reverse transcriptase (Takara Bio Inc, Shiga Japan). Real-time PCR was performed using TB Green^®^Premix Ex Taq GC (Takara Bio Inc, Shiga Japan) with a DICE Thermal cycler (Takara Bio Inc.), according to the manufacturer’s instructions. Primer sets used to detect various transcripts are described below [12, 13].

*β-Actin*-forward: 5′-TGAGAGGGAAATCGTGCGTGAC-3′

*β-Actin*-reverse: 5′-AAGAAGGAAGGCTGGAAAAGAG-3′

*OC-STAMP*-forward: 5′-ATGAGGACCATCAGGGCAGCCACG-3′

*OC-STAMP*-reverse: 5′-GGAGAAGCTGGGTCAGTAGTTCGT-3′

*DC-STAMP*-forward: 5′-TCCTCCATGAACAAACAGTTCCAA-3′

*DC-STAMP*-reverse: 5′-AGACGTGGTTTAGGAATGCAGCTC-3′

*NFATc1*-forward: 5′-CAAGTCCTCACCACAGGGCTCACTA-3′

*NFATc1*-reverse: 5′-GCGTGAGAGAGGTTCATTCTCCAAGT-3′

*Ctsk*-forward: 5′-ACGGAGGCATTGACTCTGAAGATG-3′

*Ctsk*-reverse: 5′ -GGAAGCACCAACGAGAGGAGAAAT-3′

### Statistical analysis

All quantified data are shown as means ± SD. Statistical significance of differences between groups was evaluated using Student’s *t* test, a Mann–Whitney* U* test or a one-way analysis of variance (ANOVA) using statistical software (version 25; SPSS Inc., Chicago, IL, USA) (*P < 0.05; **P < 0.01; ***P < 0.001; NS, not significant, throughout the paper).

## Results

### Establishment of DC-STAMP/OC-STAMP double knockout (DKO) mice

**Fig. 1:**
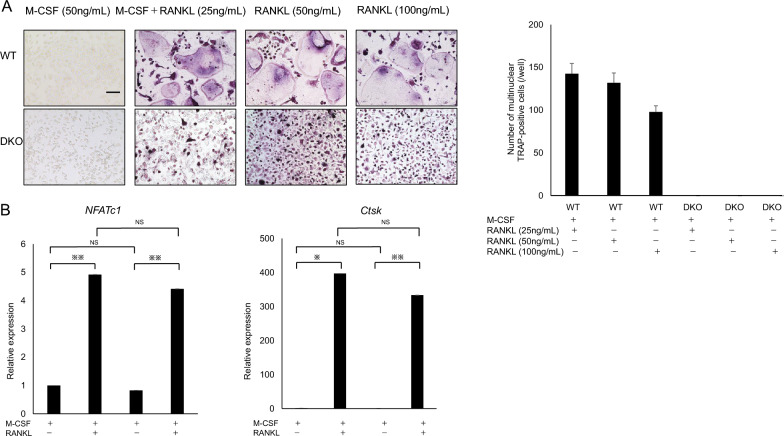
Establishment and characterization of DC-STAMP/OC-STAMP doubly deficient mice. **A** M-CSF-dependent osteoclast progenitor cells were isolated from wild-type (WT) or DC-STAMP/OC-STAMP doubly deficient (DKO) mice and cultured 5 days in indicated concentrations of M-CSF and RANKL. Osteoclast formation was evaluated by TRAP staining (left), and the number of multi-nuclear TRAP-positive cells/well was scored. Data represent mean number of multi-nuclear TRAP-positive cells/well ± SD (*n* = 3). Bar = 100 μm. **B** M-CSF-dependent osteoclast progenitor cells isolated from WT or DKO mice were cultured 5 days in the presence of M-CSF (50 ng/mL) or M-CSF (50 ng/mL) + RANKL (25 ng/mL), and expression of osteoclast differentiation markers was analyzed by real-time PCR. Data represent mean *NFATc1* or *Ctsk* expression relative to *Actb* ± SD (*n* = 3, *P < 0.05; **P < 0.01; *NS* not significant by ANOVA)

Double knockout (DKO) mice were established by crossing DC-STAMP KO with OC-STAMP KO mice (Supplementary Fig. 1). We then isolated osteoclast and FBGC common progenitor cells from WT or DKO mice and cultured them in the presence of M-CSF with or without RANKL (25 ng/ml). Although, multi-nuclear TRAP-positive osteoclasts formed only from WT cells in the presence of RANKL, DKO cells gave rise to only mono-nuclear TRAP-positive cells, as seen in either DC-STAMP KO or OC-STAMP KO cells (Fig. 1A). Real-time PCR analysis of DKO cells revealed that the expression of either DC-STAMP or OC-STAMP was below the detection limits (Supplementary Fig. 1), indicating successful establishment of the DKO model.

RANKL is a cytokine essential for osteoclast differentiation [14], and its differentiation capacity increases with concentration [15]. However, we found that experimentally increasing RANKL levels did not induce cell fusion in osteoclast progenitors derived from DKO mice, which remained mono-nuclear at all RANKL concentrations (Fig. 1A). We also observed no difference in expression of either *nuclear factor of activated T cells 1* (*NFATc1*) or *Cathepsin K*, both osteoclastic genes [16, 17], between mono-nuclear osteoclasts from DKO mice and multi-nuclear osteoclasts from WT mice (Fig. 1B). These findings suggest that in cells derived from DKO mice, differentiation to mono-nuclear osteoclasts proceeds normally but cell–cell fusion is specifically blocked.

**Fig. 2:**
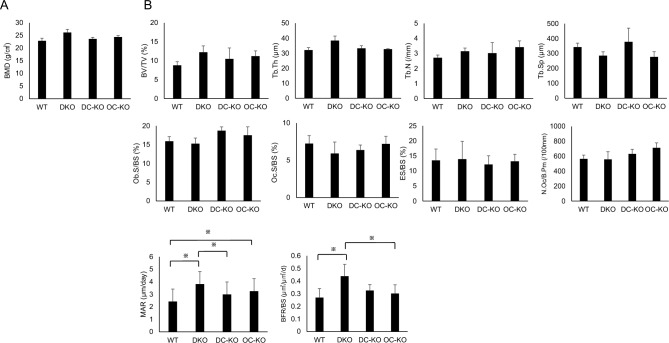
Bone mineral density and bone morphometric analysis of WT, DC-STAMP-deficient, OC-STAMP-deficient, and DKO mice. **A** Bone mineral density (BMD, mg/cm^2^) of femurs from 8-week-old wild-type (WT), DC-STAMP KO (DC-KO), OC-STAMP KO (OC-KO), and DKO mice, as analyzed by DXA. Data represent mean BMD ± SD (*n* = 5). (B) Bone morphometric analysis was performed in 8-week-old WT, DC-STAMP KO (DC-KO), OC-STAMP KO (OC-KO), and DKO mice to evaluate bone volume/tissue volume (BV/TV), trabecular thickness (Tb.Th), trabecular number (Tb.N), trabecular separation (Tb.Sp), osteoblast surface/bone surface (Ob.s/BS), osteoclast surface/bone surface (Oc.s/BS), eroded surface/bone surface (ES/BS), and number of osteoclasts (N.Oc/B.Pm per 100 mm), (*n* = 5, *P* < 0.05 by ANOVA). Also shown are mineral apposition rate (MAR) and analysis of bone formation rate/bone surface (BFR/BS). Data represent mean each parameter ± SD (WT, DC-KO, OC-KO, *n* = 5 each; DKO, *n* = 4, *P < 0.05 by ANOVA)

DXA and bone morphometric analysis in 8-week-old mice revealed that, compared to WT mice, DKO mice exhibited a trend toward increasing bone mass (Fig. 2A), although that trend was not significant (Fig. 2B and Supplementary Fig. 2). However, although bone-resorbing parameters were similar among genotypes (Fig. 2B and Supplementary Fig. 3A), DKO mice exhibited significantly increased bone formation capacity relative to WT mice, based on MAR and BFR analysis (Fig. 2B and Supplementary Fig. 3B).

**Fig. 3:**
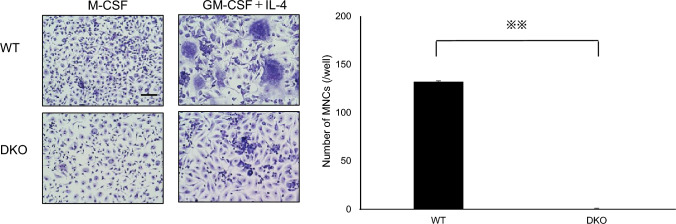
Formation of multi-nuclear foreign body giant cells is blocked in DKO mice. M-CSF-dependent progenitor cells were isolated from wild-type (WT) or DKO mice, cultured 3–4 days in the presence of M-CSF (50 ng/mL) or GM-CSF (50 ng/mL) + IL-4 (50 ng/mL) and then evaluated for formation of foreign body giant cells (FBGCs) by May–Grunwald Giemsa staining (left). The number of multi-nuclear FBGCs/well was scored (right). Data represent mean number of multi-nuclear FBGCs/well ± SD (*n* = 3, **P < 0.01 by ANOVA). Bar = 100 μm

Next, we induced FBGCs in osteoclasts/FBGC common progenitor cells derived from WT or DKO mice and stained them with May–Grunwald Giemsa (Fig. 3). As seen in osteoclast culture, although WT cells gave rise to a large number of multi-nuclear FBGCs, we observed no induction of cell–cell fusion in DKO cells, which remained mono-nuclear in FBGC culture (Fig. 3).

### Co-cultivation of DKO with WT cells induces cell–cell fusion in either osteoclasts or FBGCs

**Fig. 4:**
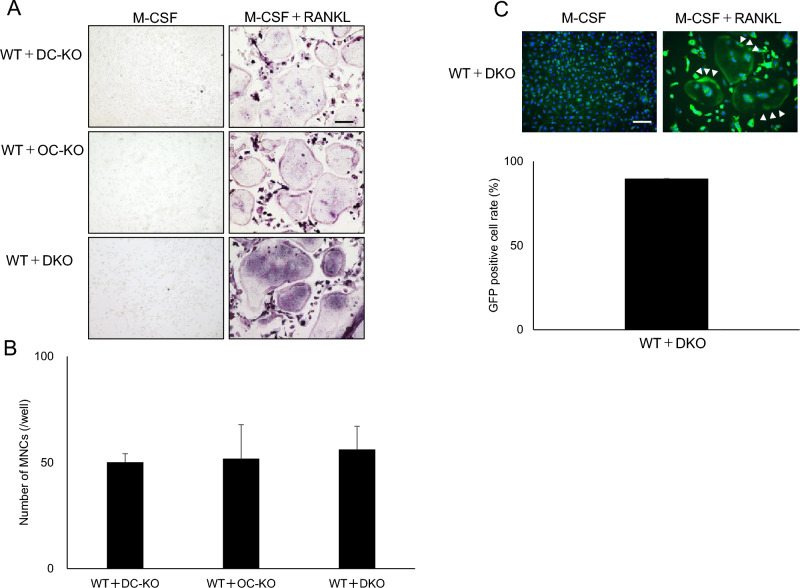
DKO cells can fuse with wild-type (WT) cells in osteoclast culture conditions. **A** and **B** Osteoclast/FBGC progenitor cells derived from WT mice were mixed with corresponding cells from either DC-STAMP KO (DC-KO), OC-STAMP KO (OC-KO) or DKO mice and cultured 4 days in the presence of M-CSF (50 ng/mL) or M-CSF (50 ng/mL) + RANKL (25 ng/mL). Osteoclast formation was evaluated by TRAP staining (**A**) and then scoring the number of multi-nuclear TRAP-positive cells per well (**B**). **C** (upper) Osteoclast/FBGC progenitor cells derived from WT mice and corresponding cells from DKO mice were mixed and cultured 5 days in the presence of M-CSF (50 ng/mL) + RANKL (25 ng/mL), stained with rabbit anti-EGFP antibody followed by Alexa488-conjugated anti-rabbit IgG antibody, and observed under a fluorescence microscope. Nuclei were stained with DAPI. Arrowheads indicate EGFP-positive multi-nuclear osteoclasts. (lower) Graph shows mean percentage of multi-nuclear GFP-positive cells among all multi-nuclear cells ± SD. Bar = 100 μm

If ligand–receptor interaction between DC-STAMP and OC-STAMP induces cell–cell fusion, then co-cultures of osteoclasts/FBGC common progenitor cells derived from WT mice with those from DKO mice should not exhibit multi-nuclear osteoclast formation. To confirm this, we mixed osteoclasts/FBGC common progenitor cells derived from WT mice with comparable cells from DC-STAMP KO, OC-STAMP KO, or DKO mice and co-cultured them for 5–6 days. Interestingly, we observed fusion and multi-nuclear osteoclast formation in co-cultures of osteoclasts/FBGC common progenitor cells from WT and DKO mice, as we had in co-cultures of WT cells with either DC-STAMP KO or OC-STAMP KO cells (Fig. 4A and B).

DC-STAMP KO mice also harbor EGFP knock-in in the DC-SATMP locus [5]; thus, either osteoclasts or FBGCs from DKO mice are EGFP-positive. As expected, we observed EGFP-positive multi-nuclear osteoclasts in co-cultures of osteoclasts/FBGC common progenitor cells derived from WT and DKO mice (Fig. 4C), suggesting that cells derived from DKO mice fuse and become multi-nuclear in these osteoclast culture conditions.

**Fig. 5:**
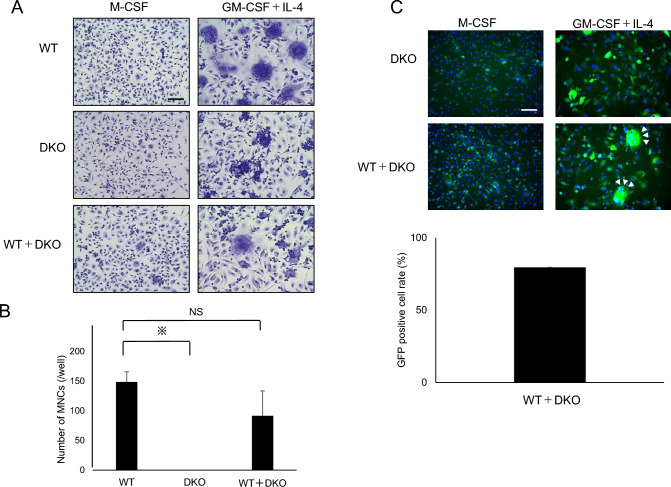
DKO cells can fuse with wild-type (WT) cells in FBGC culture conditions **A** and **B** Osteoclast/FBGC progenitor cells derived from WT or DKO mice were cultured 3 or 4 days with or without mixing in the presence of M-CSF (50 ng/mL) or GM-CSF (50 ng/mL) + IL-4 (50 ng/mL). FBGC formation was evaluated by May–Grunwald Giemsa staining (**A**), and the number of multi-nuclear FBGCs was scored (**B**). Data represent mean number of multi-nuclear FBGCs/well ± SD (*n* = 3, **P < 0.05; *NS* not significant by ANOVA). Bar = 100 μm. **C** (upper) Osteoclast/FBGC progenitor cells derived from WT or DKO mice were cultured with or without mixing in the presence of M-CSF (50 ng/mL) or GM-CSF (50 ng/mL) + IL-4 (50 ng/mL) for 4 days. Cells were then stained with rabbit anti-EGFP antibody followed by Alexa488-conjugated anti-rabbit IgG antibody. Nuclei were stained with DAPI. Arrowheads indicate EGFP-positive multi-nuclear FBGCs. (lower) Graph shows mean percentage of multi-nuclear GFP-positive cells among all whole multi-nuclear cells ± SD. Bar = 100 μm

Similarly, in FBGCs, we observed induction of multi-nuclear FBGCs in co-cultures of osteoclast/FBGC common progenitor cells from WT mice and DKO mice (Fig. 5A, B). FBGCs formed in these cultures were also EGFP-positive (Fig. 5C).

These results suggest that DC-STAMP and OC-STAMP do not induce cell–cell fusion in both osteoclasts and FBGCs in a ligand–receptor-like relationship, since DKO cells were able to fuse with WT cells.

### Co-cultures of osteoclasts/FBGC common progenitor cells derived from DC-STAMP KO and OC-STAMP KO mice do not exhibit restored cell–cell fusion

**Fig. 6:**
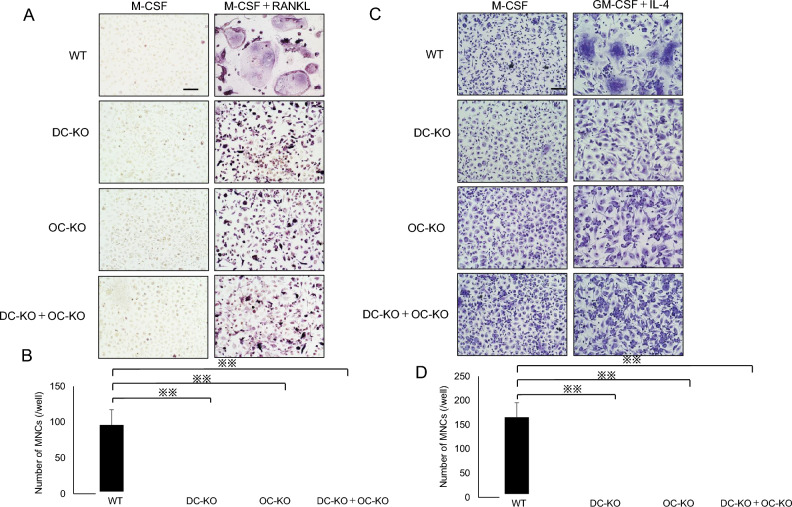
DC-STAMP KO cells co-cultured with OC-STAMP KO cells do not undergo fusion in either osteoclast or FBGC culture conditions. Osteoclast/FBGC progenitor cells derived from WT, DC-STAMP KO (DC-KO), or OC-STAMP KO (OC-KO) mice were cultured 4–5 days in the presence of either M-CSF (50 ng/mL) or M-CSF (50 ng/mL) + RANKL (25 ng/mL) (**A** and **B**) or either M-CSF (50 ng/mL) or GM-CSF (50 ng/mL) + IL-4 (50 ng/mL) (**C** and **D**). DC-KO and OC-KO cells were mixed and co-cultured 4–5 days in the presence of either M-CSF (50 ng/mL) or M-CSF (50 ng/mL) + RANKL (25 ng/mL) (**A** and **B**) or either M-CSF (50 ng/mL) or GM-CSF (50 ng/mL) + IL-4 (50 ng/mL) (**C** and **D**). Cells were then stained with TRAP for osteoclasts (**A** and **B**) or May–Grunwald Giemsa for FBGCs (**C** and **D**). **B**, **D** The number of multi-nuclear cells/well in each culture condition was scored. Data represent mean number of multi-nuclear cells/well ± SD (*n* = 3 each, **P < 0.01 by ANOVA)

Finally, to confirm that DC-STAMP and OC-STAMP induce cell–cell fusion in osteoclasts and FBGCs in a ligand–receptor-like relationship in a different way, we mixed and co-cultured osteoclasts/FBGC common progenitor cells derived from DC-STAMP KO mice with those from OC-STAMP KO mice and compared them with corresponding WT cells (Fig. 6A, C). In the case of WT cells, cell–cell fusion was induced under both osteoclast- and FBGC-induced conditions (Fig. 6B, D). On the other hand, cell–cell fusion did not occur in either osteoclast- or FBGC-induced conditions when cells derived from DC-STAMP KO or OC-STAMP KO mice were cultured alone. Interestingly, cell–cell fusion was not rescued in mixed co-cultures of DC-STAMP KO and OC-STAMP KO cells, under either osteoclast- or FBGC-inducing condition (Fig. 6B, D).

## Discussion

Multi-nucleation resulting from cell–cell fusion is a defining property of osteoclasts and considered essential for bone resorption. Formation of giant cells via cell–cell fusion is thought necessary for formation of cytoskeletal features, such as ruffled borders and actin rings, which are required for osteoclast bone resorption. However, even in DC-STAMP KO and OC-STAMP KO cells, where fusion of osteoclasts was completely abrogated, bone resorption activity was observed in mono-nuclear osteoclasts, indicating that osteoclast multi-nucleation due to fusion is not essential for bone resorption, although it may increase resorption efficiency [5, 7]. This means that there are “mono-nuclear” osteoclasts. However, the details on why does the organism provide functional molecules such as DC-STAMP and OC-STAMP to induce multi-nuclear giant cells via cell–cell fusion remain unclear.

To date, various molecules, such as ATP6v0d2, have been shown to function in osteoclast cell–cell fusion [18]. However, following the establishment of knockout mice, only DC-STAMP KO and OC-STAMP KO osteoclasts were shown to specifically and completely inhibit cell–cell fusion, without affecting mono-nuclear osteoclast differentiation [5, 7]. We found that osteoclast fusion was not induced in cultures of either DC-STAMP KO or OC-STAMP KO cells alone but was induced in co-cultures of either DC-STAMP KO or OC-STAMP KO cells with WT cells (Supplementary Fig. 4). From these findings, we initially hypothesized that cell–cell fusion is induced in DC-STAMP KO cells by interaction of OC-STAMP expressed in DC-STAMP KO cells with DC-STAMP expressed on WT cells (Supplementary Fig. 4B). Similarly, in OC-STAMP KO cells, interaction between DC-STAMP expressed in OC-STAMP KO cells and OC-STAMP expressed on WT cells likely induces osteoclast fusion (Supplementary Fig. 4C). In sperm and oocytes that undergo cell–cell fusion, fertilization is reportedly established by interaction of Izumo1 expressed in sperm with CD9 or Juno expressed in oocytes [19]. However, in our study, we found that DKO cells were able to fuse with WT cells (Supplementary Fig. 4D) and that DC-STAMP KO/OC-STAMP KO cell co-cultures did not undergo fusion (Supplementary Fig. 4F), suggesting that osteoclast cell–cell fusion is more complex than anticipated. For example, rather than DC-STAMP and OC-STAMP being expressed in separate cells and inducing cell–cell fusion via membrane ligand/receptor interaction, both DC-STAMP and OC-STAMP may become functional to induce fusion of osteoclasts and FBGCs when they are expressed in the same cell.

As previously reported, it has been suggested that deficiency of either DC-STAMP or ATP6v0d2 results in inhibition of osteoclast cell–cell fusion and upregulation of osteoblast activity [18]. The increases in MAR and BFR that we observed in DKO mice may be due to positive effects of mono-nuclear osteoclasts, rather than changes in osteoblast activity.

Currently it remains unclear how DC-STAMP and OC-STAMP function interdependently, and whether co-expression of DC-STAMP and OC-STAMP in the same cell is required for osteoclast/FBGC fusion. DC-STAMP reportedly regulated intracellular signals such as RhoA and FAK [20], and thus, both DC-STAMP and OC-STAMP may cooperate to regulate intracellular signals required for cell–cell fusion. Indeed, treatment with ROCK inhibitors reportedly promotes the formation of multi-nuclear osteoclast cells [21], although there are also reports that ROCK inhibitors block osteoclast formation in an inflammatory environment [22]. Therefore, the relationship between osteoclastogenesis and Rho/Rock signaling remains controversial.

Alternatively, cells that express DC-STAMP and OC-STAMP may initiate cell–cell fusion and function to induce fusion with recipient cells. Myoblasts were reported to fuse between myomaker and myomerger or fusion founder and fusion competent cells to form myotubes [23]. However, we found that cell–cell fusion does not occur in high-density cultures of DC-STAMP KO or OC-STAMP KO cells, even when cells are in physical contact (data not shown).

Viral infection is known to induce cell-to-cell fusion in the infected cells [24]. For example, it is known that fusion proteins expressed on the surface of virus-infected cells are multiple transmembrane molecules that form heterodimers, and that fusion of virus-infected cells proceeds via steps of cell recognition, adhesion, and fusion pore formation [25]. Structurally, both DC-STAMP and OC-STAMP are multiple transmembrane molecules [7], and they may function similarly to viral fusion proteins. If so, since DKO cells can also fuse, the presence of a third molecule that recognizes a target cell could be required. Alternatively, molecules that previously reportedly associated with cell–cell fusion may cooperate to function in cell recognition, adhesion, or fusion pore formation in osteoclasts.

Many ion channel proteins are also known to harbor multiple transmembrane domains [26]. Ions such as potassium and calcium reportedly play a role in cell–cell fusion in myoblasts [27]. Thus, the possibility remains that DC-STAMP and OC-STAMP may function as ion channels in osteoclasts and FBGCs to promote cell–cell fusion.

Taken together, our study provides new insight into mechanisms underlying cell–cell fusion of osteoclasts and FBGCs via DC-STAMP and OC-STAMP. We believe that our observations provide an essential step for further understanding of the mechanisms underlying osteoclast/FBGC fusion.

## Supplementary Information

Below is the link to the electronic supplementary material.

Supplementary file1 (PDF 3382 kb)

## Data Availability

The datasets used and/or analyzed in the current study are available from the corresponding author on reasonable request.
