# Supplementary material for: DC-STAMP and OC-STAMP cooperatively regulate osteoclast and foreign body giant cell cell–cell fusion
Source: J Bone Miner Metab. 2025 Dec 10;44(1):39–48. doi: 10.1007/s00774-025-01667-y (PMC12891103; doi:10.1007/s00774-025-01667-y)

Supplementary Figure Legends

Supplementary Figure 1. Establishment and characterization of DC-STAMP/OC-STAMP doubly-deficient mice.

M-CSF-dependent osteoclast progenitor cells isolated from WT or DKO mice were cultured 5 days in the presence of M-CSF (50ng/mL) or M-CSF (50ng/mL) + RANKL (25ng/mL), and then analyzed for *DC-STAMP* or *OC-STAMP* expression by Realtime PCR. Data represent mean *DC-STAMP* or *OC-STAMP* expression relative to *Actb* ± SD (n = 3, **P < 0.01; NS, not significant by ANOVA).

Supplementary Figure 2.

Toluidine blue staining of tibial sections from eight-week-old WT, DC-KO, OC-KO and DKO mice. Bar=100μm.

Supplementary Figure 3.

(A) TRAP staining of tibial sections from eight-week-old WT, DC-KO, OC-KO and DKO mice. Bar=10μm. (**B)** WT, DC-KO, OC-KO and DKO mice were injected with calcein twice—at 5 days and 1 day before sacrifice. Tibial sections were prepared post-sacrifice and observed under a fluorescence microscope. Bar=100μm.

Supplementary Figure 4. Schematic showing interaction of DC-STAMP with OC-STAMP in osteoclast/FBGC progenitor cells.

Shown is potential interaction between DC-STAMP and OC-STAMP expressed on WT, DC-STAMP KO (DC-KO) or OC-STAMP KO (OC-KO) osteoclast/FBGC progenitor cells.

Supplementary Figure1

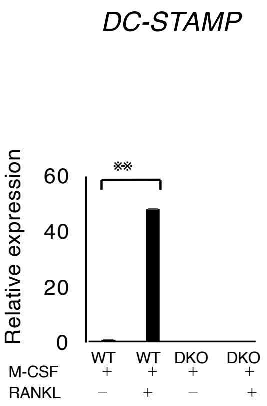

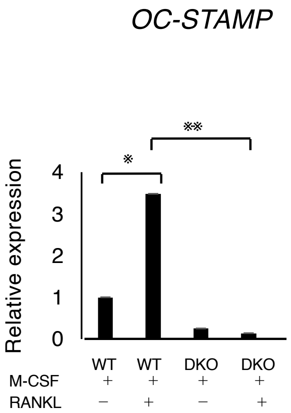

Supplementary Figure2

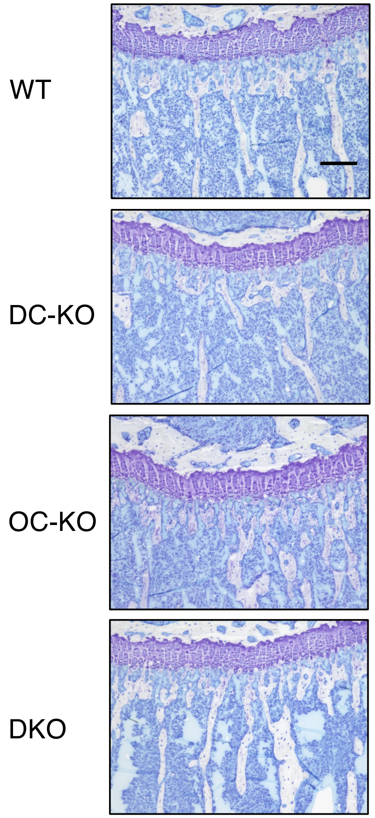

Supplementary Figure3

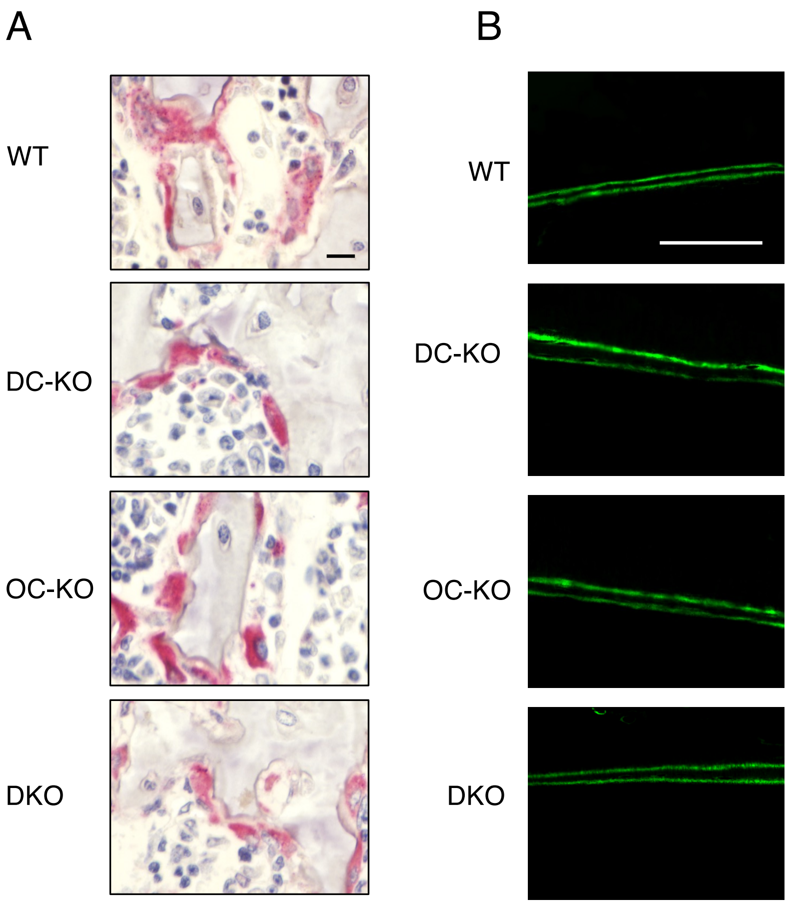

Supplementary Figure4

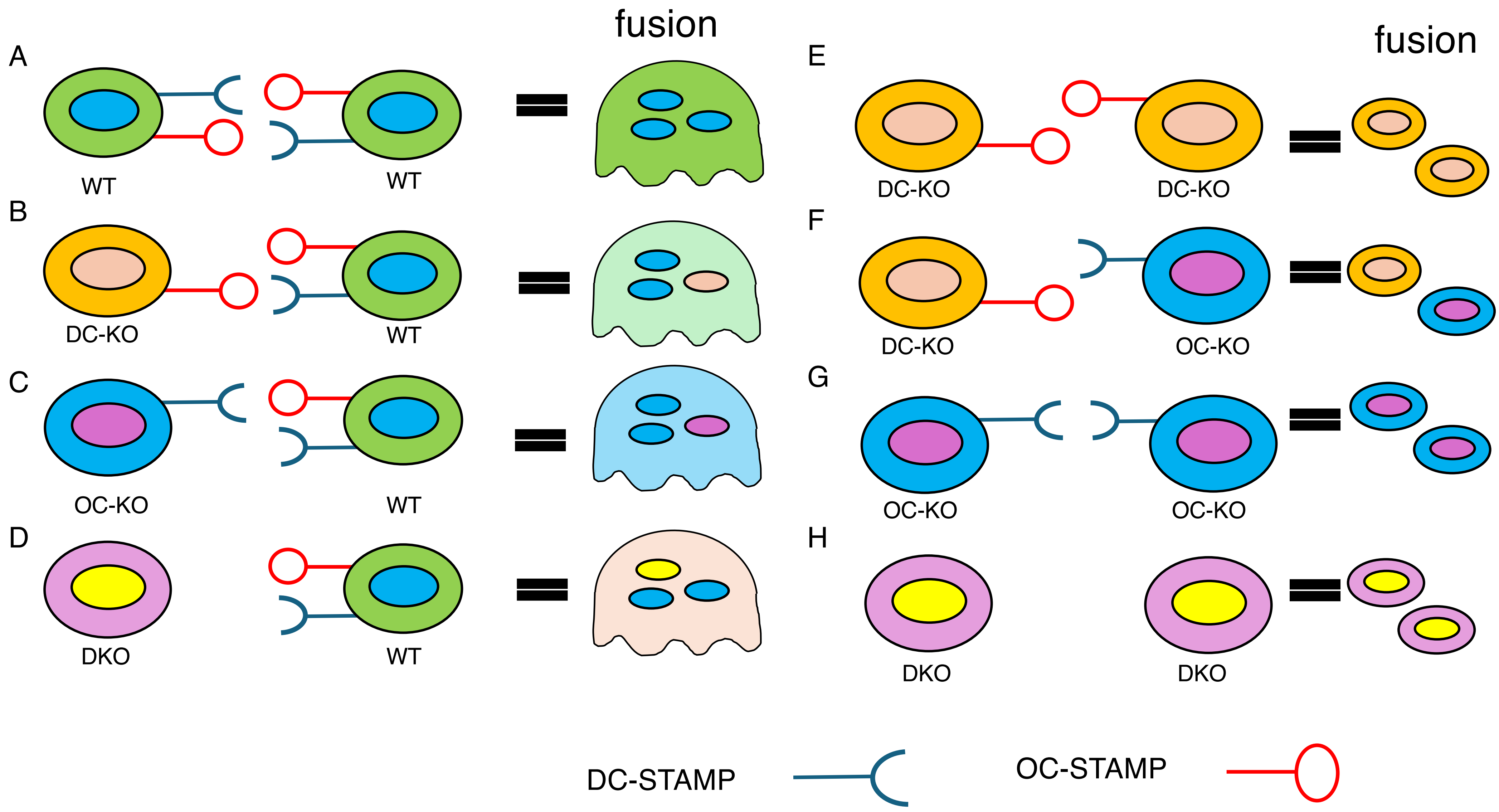

Supplement: Supplementary file 1 — Supplementary file1 (PDF 3382 kb) [file 774_2025_1667_MOESM1_ESM.pdf]
